# Clinical and molecular characterization of cystinuria in a French cohort: relevance of assessing large‐scale rearrangements and splicing variants

**DOI:** 10.1002/mgg3.294

**Published:** 2017-05-16

**Authors:** Pascaline Gaildrat, Said Lebbah, Abdellah Tebani, Bénédicte Sudrié‐Arnaud, Isabelle Tostivint, Guillaume Bollee, Hélène Tubeuf, Thomas Charles, Aurelia Bertholet‐Thomas, Alice Goldenberg, Frederic Barbey, Alexandra Martins, Pascale Saugier‐Veber, Thierry Frébourg, Bertrand Knebelmann, Soumeya Bekri

**Affiliations:** ^1^ Inserm U1245 UNIROUEN Normandie Univ Normandy Centre for Genomic and Personalized Medicine Rouen France; ^2^ Department of Nephrology Necker Hospital Paris France; ^3^ Department of Metabolic Biochemistry Rouen University Hospital Rouen France; ^4^ Department of Nephrology La Pitié‐Salpêtrière Hospital Paris France; ^5^ Interactive Biosoftware Rouen France; ^6^ Department of Urology La Milétrie Hospital Poitiers France; ^7^ Department of Pediatrics Lyon University Hospital Lyon France; ^8^ Department of Genetics Rouen University Hospital Rouen France; ^9^ Department of Transplantation CHUV Department of Pediatrics Lausanne University Hospital Lausanne Switzerland

**Keywords:** Cystinuria, exonic splicing regulatory elements, large‐scale rearrangements, *SLC3A1*, *SLC7A9*, splicing mutations

## Abstract

**Background:**

Cystinuria is an autosomal recessive disorder of dibasic amino acid transport in the kidney and the intestine leading to increased urinary cystine excretion and nephrolithiasis. Two genes, *SLC3A1* and *SLC7A9*, coding respectively for rBAT and b0,+AT, account for the genetic basis of cystinuria.

**Methods:**

This study reports the clinical and molecular characterization of a French cohort including 112 cystinuria patients and 25 relatives from 99 families. Molecular screening was performed using sequencing and Quantitative Multiplex PCR of Short Fluorescent Fragments analyses. Functional minigene‐based assays have been used to characterize splicing variants.

**Results:**

Eighty‐eight pathogenic nucleotide changes were identified in *SLC3A1* (63) and *SLC7A9* (25) genes, of which 42 were novel. Interestingly, 17% (15/88) and 11% (10/88) of the total number of variants correspond, respectively, to large‐scale rearrangements and splicing mutations. Functional minigene‐based assays were performed for six variants located outside the most conserved sequences of the splice sites; three variants affect splice sites, while three others modify exonic splicing regulatory elements (ESR), in good agreement with a new *in silico* prediction based on ΔtESRseq values.

**Conclusion:**

This report expands the spectrum of *SLC3A1* and *SLC7A9* variants and supports that digenic inheritance is unlikely. Furthermore, it highlights the relevance of assessing large‐scale rearrangements and splicing mutations to fully characterize cystinuria patients at the molecular level.

## Introduction

Cystinuria (OMIM #220100) is an inherited autosomal recessive aminoaciduria due to pathogenic variants in the *SLC3A1* (solute carrier family 3 member 1 OMIM #104614) or *SLC7A9* (solute carrier family 7 member 9 OMIM # 604144) genes, encoding respectively for rBAT and b0, +AT. These proteins are subunits of a dibasic amino acid transporter, named B0. This transporter is expressed as a stable tetramer (Reig et al. 2002) in the renal proximal tubule and the intestinal mucosa (Palacin et al. 2005). Tubular transport deficiency induces an abundant urinary excretion of dibasic amino acids, cystine, ornithine, arginine, and lysine. Cystine is poorly soluble and its crystallization results in calculi. Thus, nephrolithiasis is the only clinical expression of cystinuria. The global incidence of cystinuria is estimated at 1 per 7,000 births and varies according to geographical location (Chillaron et al. 2010). Cystinuria is characterized by a high rate of nephrolithiasis recurrence, which exceeds 90% in the absence of medical management and is up to 60% in treated patients. Adequate medical management aims i) to remove calculi and ii) to prevent cystine crystallization by increasing urine pH which preserves renal function (Prezioso et al. 2015).

Dello Strologo et al. (Dello Strologo et al. 2002) proposed a molecular classification based on the identification of pathogenic variants in the *SLC3A1* and *SLC7A9* genes. According to this classification, type A cystinuria is caused by biallelic variations that affect the *SLC3A1* gene (genotype AA), type A heterozygote individuals have normal urinary amino acid excretion; type B cystinuria is due to pathogenic variants in both *SLC7A9* alleles (genotype BB), type B heterozygote individuals may have normal or enhanced urinary excretion of dibasic amino acid; type AB (genotype AB) has been proposed with a possible digenic inheritance (Dello Strologo et al. 2002). However, recent reports demonstrated that patients with an AB genotype have a third variant leading to genotype AAB or ABB (Font‐Llitjos et al. 2005; Chillaron et al. 2010). One hundred fifty‐one and 103 variants are reported in *SLC3A1* and *SLC7A9* genes, respectively, in the public Human Genome Mutation Database accessed on October 2016 (Stenson et al. 2012). Some pathogenic variants are commonly found such as c.808C>T ‐ p.Arg270*(Pras et al. 1995), c.1400T>C ‐ p.Met467Thr (Calonge et al. 1994), c.647C>T ‐ p.Thr216Met in *SLC3A1* gene, and c.313G>A ‐ p.Gly105Arg and c.1445C>T ‐ p.Pro482Leu in *SLC7A9* gene (Chillaron et al. 2010).

Of note, large‐scale rearrangements (deletions and duplications) represent a significant proportion of *SLC3A1* and *SLC7A9* mutated alleles in cystinuria patients ranging from 11% (Bisceglia et al. 2010) to 33% (Barbosa et al. 2011). The genomic deletions of *SLC3A1* region may involve neighboring genes (*PREPL*,* CAMKMT* (previous name *C2orf34*), and *PPMB1*).

Patients with hypotonia‐cystinuria syndrome present with cystinuria, neonatal seizures, developmental delay and dysmorphic features and molecular studies allow the identification of homozygous large deletion of *SLC3A1* locus (Martens et al. 2007).

A prenatal presentation has been characterized with an ultrasound exam showing a hyperechogenicity of the colon secondary to cystine crystal deposition (Brasseur‐Daudruy et al. 2006). This finding may be related to the increase in cystine concentration in the amniotic fluid which is subsequently swallowed by the fetus.

*SLC3A1* and *SLC7A9* variants responsible for splicing alterations have been previously reported as pathogenic mutations. Indeed, among the 254 *SLC3A1* and *SLC7A9* variants listed in public Human Gene Mutation Database (HGMD^®^, 2017), 23 are considered as splicing mutations. The large majority of them are located in the most conserved intronic sequences of the 5′ or 3′ splice sites, that is, −2, −1/+1, +2 and are today systematically classified as pathogenic mutations. So far, if these specific dinucleotides are excluded, very few intronic or exonic *SLC3A1* and *SLC7A9* variants have been experimentally shown to induce an alteration of 5′ or 3′ splice sites (Pras et al. 1998; Font‐Llitjos et al. 2005; Barbosa et al. 2011) or to be responsible for a modification of exonic splicing regulatory elements (ESR) (Kummer et al. 2014).

Molecular studies of cystinuria have been reported in patients from many countries in Europe except France. In this study, we aimed to characterize at clinical and molecular levels, a French cohort of patients with cystinuria. We hypothesized that large‐scale rearrangements and splicing variants may account for a significant number of pathogenic variants. Thus, Quantitative Multiplex PCR of Short Fluorescent Fragments (QMPSF) analyses and functional minigene‐based assays have been used to characterize these specific variants.

## Material and Methods

### Recruitment of patients

A total of 112 patients and 25 relatives from 99 families with cystinuria were investigated.

Clinical data were recorded through a questionnaire sent to adult and pediatric nephrology and urology departments. The questionnaire addressed age at first symptoms, stone recurrence rates, metabolic evaluations, medical therapy, and follow‐up. Written informed consents were obtained either from the adult patients or from the parents when the patient was under 18 years old. All patients included in this study were seen at the outpatient clinic by a clinical geneticist and/or a nephrologist.

### Clinical and biochemical diagnoses

Clinical diagnosis was confirmed by quantitation of urinary amino acid and calculi identification when possible.

### Nomenclature

Nucleotide numbering is based on the cDNA sequence of *SLC3A1* (NM_000341.3) and *SLC7A9* (NM_014270.4), with c.1 denoting the first nucleotide of the translation initiation codon, as recommended by the Human Genome Variation Society.

### Molecular characterization

Probands and their relatives were analyzed for variants in *SCL3A1* and *SLC7A9* genes. Variant screening was performed by Sanger sequencing. Quantitative Multiplex PCR of Short Fluorescent Fragments analysis (QMPSF) was implemented for the detection of large‐scale rearrangements.

#### DNA extraction and sequencing analyses

Genomic DNA was extracted from venous blood using QIAamp DNA Blood Mini Kit^®^ Qiagen and was amplified *in vitro* by PCR. Multiple pairs of primers were synthesized to amplify each of *SLC3A1 and SLC7A9* exonic regions, including intron/exon boundaries and promoter (primers sequences are available upon request).

PCR reactions were carried out in 1X Thermo Scientific Buffer IV, 1.5 mm MgCl_2_, 100 μm dNTPs, 0.025 U/μL taq polymerase (Thermo Scientific), 0.6 μm of each primer.

Touchdown PCR consisted of one cycle of 95°C for 5 min for the initial denaturation step followed by 12 cycles of denaturation at 95°C for 25 sec, varying annealing (60‐48°) for 25 sec, and extension at 72°C. Then, 35 cycles were performed as follows: denaturation at 95° for 25  sec, annealing at 48° for 25 sec and extension at 72°C for 25 sec. PCR was terminated after a final cycle at 72°C for 5 min.

Direct PCR product sequencing of *SLC3A1* and *SLC7A9* genes, was performed with an ABI prism Bigdye Terminator cycle Sequencing Ready Reaction Kit (PE Applied Biosystem/and ABI model 3130xl Genetic Analyser). Patients’ genomic sequences comparison with the reference sequence was done by Variant Reporter software (Applied Biosystem).

#### QMPSF analyses

Short exonic fragments were simultaneously amplified by PCR and distributed across seven multiplex reactions for *SLC7A9*,* PPM1B*,* SLC3A1*,* PREPL*, and *CAMKMT* genes (primer sequences are available upon request); 38 amplicons with a size between 140 and 306 bp were designed to cover these gene sequences. One additional autosomic fragment, corresponding to the exon 13 of the *HMBS* gene located on chromosome 11, was coamplified, as a control PCR. A fragment from chromosome X was coamplified as quantification control (exon 8 of the *NHS* gene or exon 4 of the *MECP2* gene). The PCR reaction was performed in a 25 μL final volume containing 100 ng of genomic DNA, 0.3–0.9 mm of each primer, 200 mm dNTPs, 25 mm MgCl2, 10% DMSO, 1X Thermo Scientific Buffer IV, and 1U of Taq DNA polymerase (ABgene, Courtaboeuf, France). The PCR consisted of 25 cycles of 94°C for 15 sec, 50°C for 15 sec, and 72°C for 15 sec, preceded by a 5‐min initial denaturation step at 94°C and followed by a 5‐min final extension at 72°C. One μL of the PCR product was resuspended in a mix containing 10 μL of deionized formamide, 0.5 μL of GeneScan‐500 Rox (PE Applied Biosystems, Foster City, CA, USA). After denaturation for 2 min 30 sec at 94°C, 2 μL of each sample was loaded on an Applied Biosystems model 3130xl Genetic Analyzer automated sequencer (PE Applied Biosystems). Data were analyzed using the Genescan software (PE Applied Biosystems,). Electropherograms were superimposed to those generated from a normal control DNA by adjusting the peaks obtained for the control amplicon and the heights of the corresponding peaks to the same level. Peaks were then compared between the different samples.

### *In silico* analysis of the variants

#### Allele frequency analysis

The frequency in human population of the novel single‐nucleotide variants identified in this study was evaluated using different databases (Exome Aggregation Consortium (Lek et al. 2016), Exome Sequencing Project (ESP) (Auer et al. 2016), the 1000 Genomes Project (Auton et al. 2015), and the dbSNP (Kitts et al. 2013). Only the variants with frequency below 1% were considered for further analyses.

#### Computational predictions for the functional impact of missense variants

The potential functional impact on the protein of novel missense variants with low frequency were then evaluated using *in silico* methods, such as Align GVGD (Tavtigian et al. 2008) SIFT (Kumar et al. 2009), PolyPhen2 (Adzhubei et al. 2010), and MutationTaster (Schwarz et al. 2010).

#### Bioinformatics predictions of splicing variants

The *in silico* tool MaxEntScan (Yeo and Burge 2004) was used to predict variant‐induced alterations of 3′ and 5′ splice sites. This algorithm was interrogated using the integrated software Alamut (Interactive Biosoftware, France). One newly developed bioinformatics approach was used to evaluate the potential impact of variants on exonic splicing regulatory elements (ESR). This method is based on the reported quantitative evaluation of all RNA hexamers as potential ESR (Ke et al. 2011), which allows the calculation of total ESRseq score changes (ΔtESRseq) for each variant, as previously described (Di Giacomo et al. 2013; Soukarieh et al. 2016).

### Functional splicing minigene reporter assay

**Figure 1:**
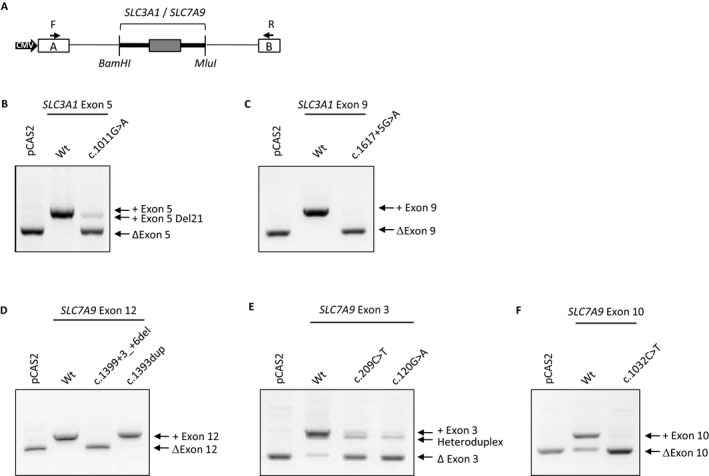
Effect on splicing of selected variants located in *SLC3A1* and *SLC7A9* genes, assessed using a functional minigene assay. (A) Schematic representation of the pCAS2‐*SLC3A1*/*SLC7A9* minigene used in the splicing reporter assay. Boxes indicate exons, whereas lines in between represent introns. The minigenes were generated by inserting a genomic fragment, containing the *SLC3A1* or *SLC7A9* exon of interest (gray box), as well as part of the upstream and downstream flanking intronic sequences (thick lines), into the intron of the minigene using the BamHI and MluI restriction sites. Expression of the minigenes is driven by the CMV promoter. Arrows above the minigene exons A and B (white boxes) indicate the positions of primers used in RT‐PCR analysis. (B–F) Analysis of the splicing pattern of the wild‐type and mutant pCAS2‐*SLC3A1*/*SLC7A9* minigenes for the selected variants. Wild‐type (WT) and mutant pCAS‐2 *SLC3A1*/*SLC7A9* constructs were transiently expressed in HeLa cells by transfection. The splicing patterns of the minigene transcripts were then analyzed by RT‐PCR as described under Materials and Methods. The image shows the electrophoresis on a 2% agarose ethidium bromide‐stained gel of the RT‐PCR products obtained for each minigene. The identities of the RT‐PCR products, with the inclusion (+) or the skipping (Δ) of the exon are indicated on the right.

In order to assess the impact on splicing of *SLC3A1* and *SLC7A9* variants, functional minigene‐based assays were performed, as previously described (Tournier et al. 2008; Gaildrat et al. 2010) (Fig. 1A). Briefly, the *SLC3A1* or *SLC7A9* exons relevant to this study along with approximatively 150 bp of flanking intronic sequences were amplified by PCR from patient genomic DNA using forward and reverse primers carrying 5′ tails that contained BamHI and MluI restriction sites, respectively (primer sequences are available upon request). After digestion with BamHI and MluI restriction enzymes, the PCR products were inserted into the intron of the splicing reporter minigene vector pCAS2 (Fig. 1A). This vector carries two exons (named A and B) with a sequence derived from the human SERPING1/C1NH gene, separated by an intron with BamHI and MluI cloning sites (Tournier et al. 2008; Gaildrat et al. 2010; Soukarieh et al. 2016). Expression of the pCAS2 minigene is under the control of a CMV promoter. The inserts of all minigene constructs were sequenced to verify the presence of the variants and to ensure that no extra mutations were introduced during the cloning process.

HeLa cells were obtained from ATCC and grown in Dulbecco's modified Eagle's medium (Life Technologies), supplemented with 10% fetal calf serum, under a 5% CO_2_ atmosphere at 37°C. Next, WT and mutant pCAS2 minigene plasmids were transiently transfected into HeLa cells using the FuGENE 6 transfection reagent (Roche Applied Science). Total RNA was then extracted 24 h posttransfection using the NucleoSpin RNA II kit (Macherey Nagel), according to the manufacturer's instructions, including a DNase I treatment. The splicing pattern of each minigene was analyzed by RT‐PCR (30 cycles of amplification) from 200 ng total RNA in a 25 μL reaction volume, using the OneStep RT‐PCR kit (Qiagen), with the primers pCASKO1F (5′‐TGACGTCGCCGCCCATCAC‐3′) and pCAS2R (5′‐ATTGGTTGTTGAGTTGGTTGTC‐3′), respectively, located in exons A and B of the minigene pCAS2 (Fig. 1A). RT‐PCR products were separated by electrophoresis on 2% agarose gels containing ethidium bromide and visualized by exposure to ultraviolet light under nonsaturating conditions using the Gel Doc XR image acquisition system (Bio‐RAD). Gel extraction and sequencing of the RT‐PCR products were carried out as previously described [Gaildrat et al. 2010].

### Statistical analysis

As a first step, we conducted a descriptive analysis of the population with the means and medians for quantitative variables and frequencies for qualitative variables. In a second step, a univariate analysis was performed to determine what might be the factors influencing genotype–phenotype correlation. For this, we used the Wilcoxon test, test of comparison of two observed medians. In a third step, we compared our results, with those of three recent European studies on genotype distribution (Chatzikyriakidou et al. 2008; Bisceglia et al. 2010; Wong et al. 2015) and with those of a meta‐analysis on recurrent variant frequency (Chillaron et al. 2010). A Person's Chi‐squared test was performed to compare two observed frequencies or Fisher's exact test if the sample sizes required.

An alpha risk of 5% and a significant level of *P* < 0.05 were defined for each statistical test.

All statistical analyses were performed using Excel 2007 and the R statistical software.

## Results

### Identification of 42 novel pathogenic variants in *SLC3A1* and *SLC7A9* genes

This study reports the clinical and molecular characterization of a French cohort including 112 cystinuria patients and 25 relatives from 99 families. We identified 115 variants in *SLC3A1* (69) and *SLC7A9* (46) genes. A variant was considered as not pathogenic if it exhibits an allele frequency above 1% in human population. Based on this criterion, 24 variants were considered as benign (Table S1). Besides, in our cohort, the variant *SLC3A1* c.231T>A – p.(=) has always been identified in combination with the pathogenic variant *SLC3A1* c.1400T>C ‐ p.Met467Thr in 25 patients. The complete linkage of these two variants has been previously described in other populations (Gasparini et al. 1995; Harnevik et al. 2001).

The remaining 90 variants included 8 nonsenses, 14 small deletions/insertions, 15 large‐scale rearrangements, 4 intronic variants located in the most conserved sequences of splice sites (‐2,‐1/+1,+2), 44 missenses, and 5 variants of unknown significance (VUS) (three synonymous and two intronic variants outside the most conserved sequences of splice sites).

Among these variants, 46 were previously reported (Tables 1 and 2). The remaining 44 novel variants included six nonsenses, nine small deletions/insertions, eight large‐scale rearrangements, two intronic variants located in the most conserved sequences of splice sites (−2,−1/+1,+2), 17 missenses, and two variants of unknown significance (VUS): 1 synonymous and 1 intronic variant outside the most conserved sequences of splice sites. Except for the missense variants and VUS, these variants were considered as pathogenic without any further analysis. For the missense variants, 15 were assumed to be deleterious based on the output of at least two of the following *in silico* algorithms, Align GVGD (Tavtigian et al. 2008), SIFT (Kumar et al. 2009), PolyPhen2 (Adzhubei et al. 2010), or MutationTaster (Schwarz et al. 2010) (Table S2). Hence, two missense variants in *SLC7A9*, c.26G>A (p.Arg9Gln) and c.397T>C (p.Ser133Pro), were classified as variants of unknown significance because they were predicted nonpathogenic by all or by three out of four algorithms, respectively. For the synonymous and intronic variants located outside the most conserved sequences of splice sites, their functional impacts on splicing were assessed using splicing minigene assays. In addition, variants previously reported as splicing mutations without functional data were included in this analysis.

**Table 1:** *SLC3A1* sequence variants

Variant type	Position	Nucleotide change	Predicted protein	Alleles	Patients	Reference
Missense	Exon 1	c.257G>A	p.(Arg86Gln)	1	1	Novel
	Exon 1	c.371A>G	p.(Tyr124Cys)	1	1	Font‐Llitjos et al. (2005)
	Exon 2	c.503C>T	p.(Ser168Leu)	1	1	Novel
	Exon 2	c.535G>T	p.(Asp179Tyr)	2	2	Skopkova, et al., (2005)
	Exon 2	c.566C>T	p.(Thr189Met)	2	2	Font‐Llitjos et al. (2005)
	Exon 2	c.595G>C	p.(Ala199Pro)	1	1	Novel
	Exon 3	c.647C>T	p.(Thr216Met)	16	10	Bisceglia et al. (2010)
	Exon 3	c.647C>G	p.(Thr216Agr)	1	1	Novel
	Exon 3	c.763T>G	p.(Trp255Gly)	2	2	Novel
	Exon 4	c.789T>G	p.(Ser263Arg)	1	1	Novel
	Exon 4	c.851A>G	p.(Asp284Gly)	1	1	Novel
	Exon 6	c.1051A>G	p.(Thr351Ala)	1	1	Novel
	Exon 6	c.1094G>T	p.(Arg365Leu)	1	1	Albers, et al., (1999)
	Exon 7	c.1144G>T	p.(Gly382Trp)	1	1	Novel
	Exon 7	c.1190A>G	p.(Tyr397Cys)	3	2	Eggermann, (2009)
	Exon 7	c.1318T>C	p.(Trp440Arg)	2	1	Novel
	Exon 8	c.1354C>T	p.(Arg452Trp)	4	4	Endsley, et al., (1997)
	Exon 8	c.1364C>T	p.(Ser455Leu)	2	2	Font‐Llitjos et al. (2005)
	Exon 8	c.1367G>A	p.(Arg456His)	2	1	Font‐Llitjos et al. (2005)
	Exon 8	c.1400T>C	p.(Met467Thr)	27	25	Calonge et al. (1994)
	Exon 8	c.1400T>A	p.(Met467Lys)	2	2	Calonge et al. (1994)
	Exon 9	c.1518G>C	p.(Lys506Asn)	2	1	Novel
	Exon 9	c.1527G>A	p.(Met509Ile)	1	1	Novel
	Exon 9	c.1529A>C	p.(Gln510Pro)	1	1	Novel
	Exon 9	c.1607T>G	p.(Val536Gly)	2	2	Gitomer, et al., (1998)
	Exon 10	c.1640C>G	p.(Ser547Trp)	1	1	Bisceglia, et al., (2001)
	Exon 10	c.1684G>C	p.(Glu562Gln)	1	1	Brauers and Eggermann, (2006)
	Exon 10	c.1701G>T	p.(Arg567Ser)	1	1	Brauers and Eggermann, (2006)
	Exon 10	c.1796T>C	p.(Phe599Ser)	1	1	Harnevik et al. (2001)
Nonsense	Exon 1	c.163C>T	p.(Gln55*)	1	1	Novel
	Exon 2	c.464T>G	p.(Leu155*)	1	1	Novel
	Exon 4	c.792G>A	p.(Trp264*	2	2	Chatzikyriakidou et al. (2008)
	Exon 4	c.808C>T	p.(Arg270*)	7	5	Pras et al. (1995)
	Exon 6	c.1134C>A	p.(Tyr378*)	1	1	Novel
	Exon 8	c.1352C>G	p.(Ser451*)	1	1	Novel
	Exon 9	c.1459G>T	p.(Gly487*)	1	1	Novel
	Exon 10	c.2011C>T	p.(Arg671*)	1	1	Novel

Variant type	Position	Variant (nucleotide change)	Predicted protein	Alleles	Patients	Reference
Small deletion	Exon 5	c.979_980del	p.(Asp327*)	1	1	Horsford, et al., (1996)
	Exon 10	c.1693_1695del	p.(Leu565del)	1	1	Novel
	Exon 10	c.1699_1700del	p.(Arg567Glyfs*9)	3	3	Font‐Llitjos et al. (2005)
	Exon 10	c.1750del	p.(Arg584Glufs*14)	1	1	Gasparini et al. (1995)
	Exon 10	c.1754_1755del	p.(Glu585Alafs*24)	1	1	Novel
Small insertion	Exon 2	c.594dup	p.(Ala199Serfs*4)	1	1	Novel
	Exon 9	c.1545_1563dup	p.(Ala522Lysfs*6)	1	1	Novel
	Exon 10	c.1744_1745insC	p.(Tyr582Serfs*28)	1	1	Novel
Deletion‐insertion	Exon 7	c.1159_1160delinsAA	p.(Ala387Lys)	2	2	Novel
Splicing	Intron 1	c.430+1G>T^a^	p.(?)^c^	1	1	Novel
	Exon 5	c.1011G>A^b^	p.(Pro337=)^c^	3	3	Saadi et al. (1998)
	Intron 8	c.1500+1G>T^a^	p.(?)^c^	3	3	Horsford, et al., (1996)
	Intron 9	c.1617+5G>A^b^	p.(?)^c^	1	1	Novel
Duplication	Exon 4 and 5	c.(765+1_766‐1)_(1011+1_1012‐1)dup	p.(?)	1	1	Novel
	Exon 5 to 9	c.(891+1_892‐1)_(1617+1_1618‐1)dup	p.(?)	17	16	Schmidt et al. (2003)
Large deletion	Exon 1 *SLC3A1* to exon 2 *CAMKMT*	*SLC3A1; NM_000341.3* c.(?_1)_*CAMKMT; NM_024766.4* c.(311+1_?)del	p.(?)	1	1	Novel
	Exon 2 and exon 3	c.(430+1_431‐1)_(765+1_766‐1)del	p.(Gly144Valfs*12)	1	1	Gitomer, et al., (1998)
	Exon 2 to exon 4	c.(430+1_431‐1)_(891+1_892‐1)del	p.(Ile145Asnfs*11)	1	1	Bisceglia et al. (2010)
	Exon 2 *SLC3A1* to exon 2 PREPL	*SLC3A1 NM_000341.3:*c.(430+1_431‐1)_*PREPL NM_006036.4:*c.(219+1_?)del	p.(?)	4	3	Jaeken, et al., (2006)
	Exon 3 to exon 9	c.(610+1_611‐1)_(1616+1_1617‐1)del	p.(Leu205Profs*22)	1	1	Novel
	Exon 4 SLC3A1 to exon 9	c.(765+1_766‐1)_(1616+1_1617‐1)del	p.(Leu256_Pro337del)	1	1	Novel
	Exon 5 SLC3A1 to exon 11 *PREPL*	*SLC3A1 NM_000341.3:*c.(?_892‐1)_*PREPL; NM_006036.4:*c.(1896+1_?)del	p.(?)	2	1	Jaeken, et al., (2006)
	Exon 7 *SLC3A1* to exon 2 *PREPL*	*SLC3A1 NM_000341.3:*c.(?_1137‐1)_*PREPL NM_006036.4:*c.(219+1_?)del	p.(?)	1	1	Novel
	Exon 8	c.(1332+1_1333‐1)_(1500+1_1501‐1)del	p.(Ile445_Ile500del)	1	1	Novel
	Exon 10 *SLC3A1* to exon 2 *PREPL*	*SLC3A1 NM_000341.3:*c.(?_1618‐1)_*PREPL NM_006036.4:*c.(219+1_?)del	p.(?)	1	1	Jaeken, et al., (2006)
	Exon 10 *SLC3A1* to exon 3 *CAMKMT*	*SLC3A1 NM_000341.3:*c.(?_1618‐1)_*CAMKMT NM_024766.4:*c.(376+1_?)del	p.(?)	1	1	Novel

a Variants located in the canonical intronic splice site sequences.

b Variants with an impact on splicing in minigene assay (this study).

c Predicted consequence at the protein level without taking into account the impact of the variant on splicing.

**Table 2:** SLC7A9 sequence variants

Mutation type	Position	Variant (nucleotide change)	Predicted protein	Alleles	Patients	Reference
Missense	Exon 3	c.131T>C	p.(Ile44Thr)	1	1	Leclerc, et al., (2002a)
	Exon 4	c.313G>A	P.(Gly105Arg)	14	11	Feliubadalo, et al., (1999)
	Exon 4	c.368C>T	p.(Thr123Met)	1	1	Font et al. (2001)
	Exon 4	c.380T>C	p.(Ile127Thr)	1	1	Novel
	Exon 5	c.508G>A	p.(Val170Met)	1	1	Feliubadalo, et al., (1999)
	Exon 5	c.511C>G	p.(Arg171Gly)	1	1	Novel
	Exon 5	c.517G>C	p.(Gly173Arg)	1	1	Lee, et al., (2010)
	Exon 5	c.544G>A	p.(Ala182Thr)	3	3	Feliubadalo, et al., (1999)
	Exon 5	c.562G>A	p.(Val188Met)	1	1	Font‐Llitjos et al. (2005)
	Exon 10	c.992C>T	p.(Ala331Val)	2	2	Botzenhart, et al., (2002)
	Exon 10	c.997C>T	p.(Arg333Trp)	2	1	Font et al. (2001)
	Exon 11	c.1166C>T	p.(Thr389Met)	1	1	Wong et al. (2015)
Small deletion	Exon 4	c.285del	P.(Glu96Serfs*5)	1	1	Novel
	Exon 4	c.414_415del	p.(Pro139Leufs*69)	1	1	Wong et al. (2015)
Small insertion	Exon 6	c.614dup	p.(Asn206Glufs*3)	5	4	Leclerc, et al., (2002a)
	Exon 12	c.1393dup	P.(Ile465Asnfs*23)	1	1	Novel
Deletion‐Insertion	Exon 3	c.91delinsTGTGAT	p.(Gly31Cysfs*61)	1	1	Novel
Splicing	Exon 3	c.120G>A^b^	p.(Val40=)^c^	1	1	Wong et al. (2015)
	Exon 3	c.209C>T^b^	p.(Ala70Val)^c^	1	1	Font et al. 2001)
	Intron 5	c.604+2T>C^a^	p.(?)^c^	1	1	Font‐Llitjos et al. (2005)
	Exon 10	c.1032C>T^b^	p.(Ile344=)^c^	1	1	Novel
	Intron 10	c.1074+2T>C^a^	p.(?)^c^	1	1	Novel
	Intron 12	c.1399+3_1399+6del^b^	p.(?)^c^	1	1	Font et al. (2001)
Large deletion	Exon 10	c.(977+1_978‐1)_(1074+1_1075‐1)del	p.(Leu327Valfs*3)	1	1	Novel
	Exon 12	c.(1224+1_1225‐1)_(1399+1_1400‐1)del	p.(Val409Serfs*10)	2	2	Font‐Llitjos et al. (2005)

a Variants located in the canonical intronic splice site sequences.

b Variants with an impact on splicing in minigene assay (this study).

c Predicted consequences at the protein level without taking into account the impact of the variant on splicing.

### Splicing effect of selected *SLC3A1* and *SLC7A9* variants

In this study, four *SLC3A1* and *SLC7A9* variants were identified at the most conserved intronic positions (−2, ‐1/+1, +2) of the splice site sequences. (Tables 1 and 2). Two of them have been previously reported, that is, *SLC3A1* c.1500+1G>T (Saadi et al. 1998) and *SLC7A9* c.604+2T>C (Font‐Llitjos et al. 2005), whereas the two others were novel, that is, *SLC3A1* c.430+1G>T and *SLC7A9* c.1074+2T>C. All of them were considered as splicing pathogenic mutations. We hypothesized that, among the variants identified in this cohort, additional variants could impact splicing. Initially, a potential effect on splice sites was predicted, using the algorithm MaxEntScan (Yeo and Burge 2004), for four variants of unknown significance identified in this study (Table 3). Indeed, *SLC3A1* c.1011G>A (p.=), *SLC3A1* c.1617+5G>A, and *SLC7A9* c.1399+3_+6del were predicted to alter reference 5′ splice sites, whereas *SLC7A9* c.120G>A (p.=) could create a new 3′ splice site. The impact on RNA splicing of these selected variants was then assessed using a functional minigene splicing assay (Fig. 1A).

**Table 3:** Bioinformatics predictions of the variant effect on splicing

Gene	Variant	Position	Splice site (MaxEntScan) (Wt → Var)	ESR (ΔtESRseq) (Var vs. Wt)	Effect on splicing (Minigene)	Predicted consequences on RNA and Protein
*SLC3A1*	c.1011G>A	Last base of exon 5	Reference 5′ ss: 6.866 → 0	n.a.	Exon 5 skipping (major)	r.892_1011del, p.Glu_Pro337del
	c.1617+5G>A	+5 intron 9	Reference 5′ ss: 9.1 → 2.3	n.a.	Exon 5 del 21 nt (minor)	r.991_1011del, p.Val331_Pro337del
					Exon 9 skipping (full)	r.1501_1617, p.Asn501_Asp539del
*SLC7A9*	c.120G>A	+33 exon 3	Creation 3′ ss: 0 → 6.6	−2.9	Exon 3 skipping (major)	r.88_235del, p.Leu30Valfs*11
	c.209C>T	−27 exon 3	No change	−1.8	Exon 3 skipping (major)	r.88_235del, p.Leu30Valfs*11
	c.225C>T^1^	−11 exon 3	No change	−2.2	Exon 3 skipping^a^	r.88_235del, p.Leu30Valfs*11
	*c*.1032C>T	−43 in exon 10	No change	−2.6	Exon 10 skipping (full)	r.978_1074del, p.Leu327Valfs*3
	c.1399+3_+6del	+3_+6 in intron 12	Reference 5′ ss : 10.1 → 0	n.a.	Exon 12 skipping (full)	r.1225_1399del, p.Val409Serfs*10

Wt, wild‐type; Var, variant; n.a., not applicable; ss, splice site; ESR, exonic splicing regulatory elements.

a Kummer et al. (2014).

In this assay, our results showed that all the processed RNA expressed from the wild‐type *SLC3A1* exon 5 minigene retained *SLC3A1* exon 5, whereas the major spliced transcripts expressed from the mutant *SLC3A1* c.1011G>A minigene exhibited the skipping of exon 5 (Fig. 1B). A second minor aberrant transcript, corresponding to the inclusion of *SLC3A1*exon 5 deleted for the last 21 bp, was also detected. These data are consistent with the bioinformatics predictions assessing the effect of *SLC3A1* c.1011G>A on splicing, suggesting that this synonymous variant, located at the last position of exon 5, alters the strength of the reference 5′ splice site (Table 3). The minor effect is predicted to be the consequence of the use of a cryptic exonic 5′ splice site, located 21 nucleotides upstream the reference 5′ splice site (data not shown).

The second tested variant, *SLC3A1* c.1617+5G>A, located in intron 9, led to total skipping of exon 9 in the minigene splicing assay (Fig. 1C). These data are consistent with the bioinformatics predictions suggesting that this variant modified the reference 5′ splice site of intron 9 **(**Table 3).

The *SLC7A9* c.1399+3_+6del variant, located in intron 12, was identified in patient P82, who also carries another variant in trans, c.1393dup, in exon 12 (Table S3). Both variants were tested in the minigene splicing assay. The results showed that all the processed RNA expressed from the wild‐type and the mutant c.1393dup *SLC7A9* exon 12 minigenes retained *SLC7A9* exon 12, while the mutant c.1399+3_+6del induced exon 12 skipping (Fig. 1D). These results are in good agreement with the *in silico* data which showed that this variant was responsible for the destruction of the reference 5′ splice site (Table 3).

The synonymous variant *SLC7A9* c.120G>A ‐ p.(=), located in exon 3, was identified in patient P96, who also carries in trans a missense variant in this exon, c.209C>T ‐ p.(Ala70Val) (Table S3). Both variants were then tested in the minigene splicing assay. This study revealed that the wild‐type *SLC7A9* exon 3 minigene generated two different transcripts: a major transcript corresponding to the normal inclusion of exon 3 and a minor transcript lacking this exon (Fig. 1E). In contrast, minigene constructs carrying either the *SLC7A9* c.120G>A or c.209C>T variants produced a major transcript without exon 3. These data were surprising because the bioinformatics predictions dedicated to splice site analysis suggested that the variant c.120G>A would create a new 3′ splice site whereas no effect was predicted for the variant c.209C>T (Table 3). The skipping of exon 3 induced by these two variants might then be the consequence of an alteration of exonic splicing regulatory elements (ESR). Interestingly, the output of a new *in silico* method based on the calculation of total ESRseq score changes (ΔtESRseq) (Ke et al. 2011; Di Giacomo et al. 2013; Soukarieh et al. 2016) supports this hypothesis. Indeed, *SLC7A9* c.120G>A and c.209C>T are associated with negative ΔtESRseq values, ‐2.9 and ‐1.8, respectively (Table 3), which suggest that these two variants induce the exon 3 skipping through the destruction of exonic splicing enhancer (ESE) and/or the creation of exonic splicing silencer (ESS).

In addition to these four variants initially selected based on splice site predictions, we also tested the impact on splicing of the variant *SLC7A9* c.1032C>T p.(=) based on its negative ΔtESRseq value (‐2.6) (Table 3), which suggested a potential impact on ESR. In the functional minigene assay, the wild‐type *SLC7A9* exon 10 minigene generated two different transcripts: a major one with exon 10 and a minor one without (Fig. 1F). The *SLC7A9* c.1032C>T variant was responsible for the major production of transcripts lacking exon 10, suggesting an effect through the alteration of ESR.

For all these variants, the predicted consequences at the RNA and protein levels of the splicing alteration were evaluated (Table 3).

### Large‐scale rearrangements in *SCL3A1* and *SLC7A9* genes

**Figure 2:**
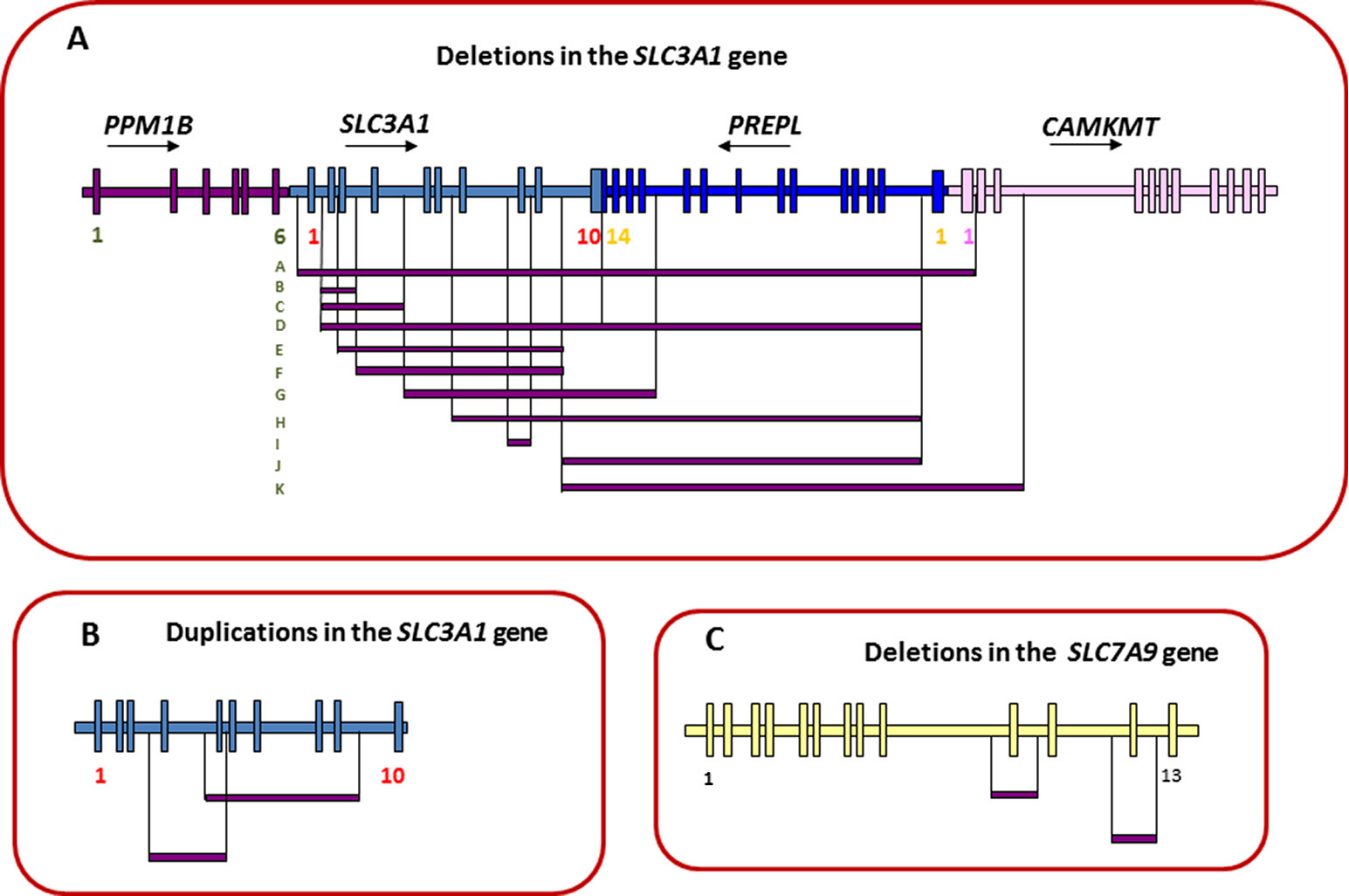
Schematic representation of large‐scale rearrangements in *SLC3A1*,*PREPL*,*CAMKMT*, and *SLC7A9* genes. Genes are written on top of the arrows indicating the direction of the transcription. The exons are indicated (squares). (A) Schematic representation of 11 deletions in the 2p21 locus. (B) Schematic representation of 11 deletions in the *SLC3A1* gene. (C) Schematic representation of two deletions in the *SLC7A9* gene.

QMPSF analyses were conducted in 50 cases and allowed the characterization of 11 deletions, 2 duplications in *SLC3A1* gene and 2 deletions in *SCL7A9* gene, 8 of which are novel (Fig. 2).

**Figure 3:**
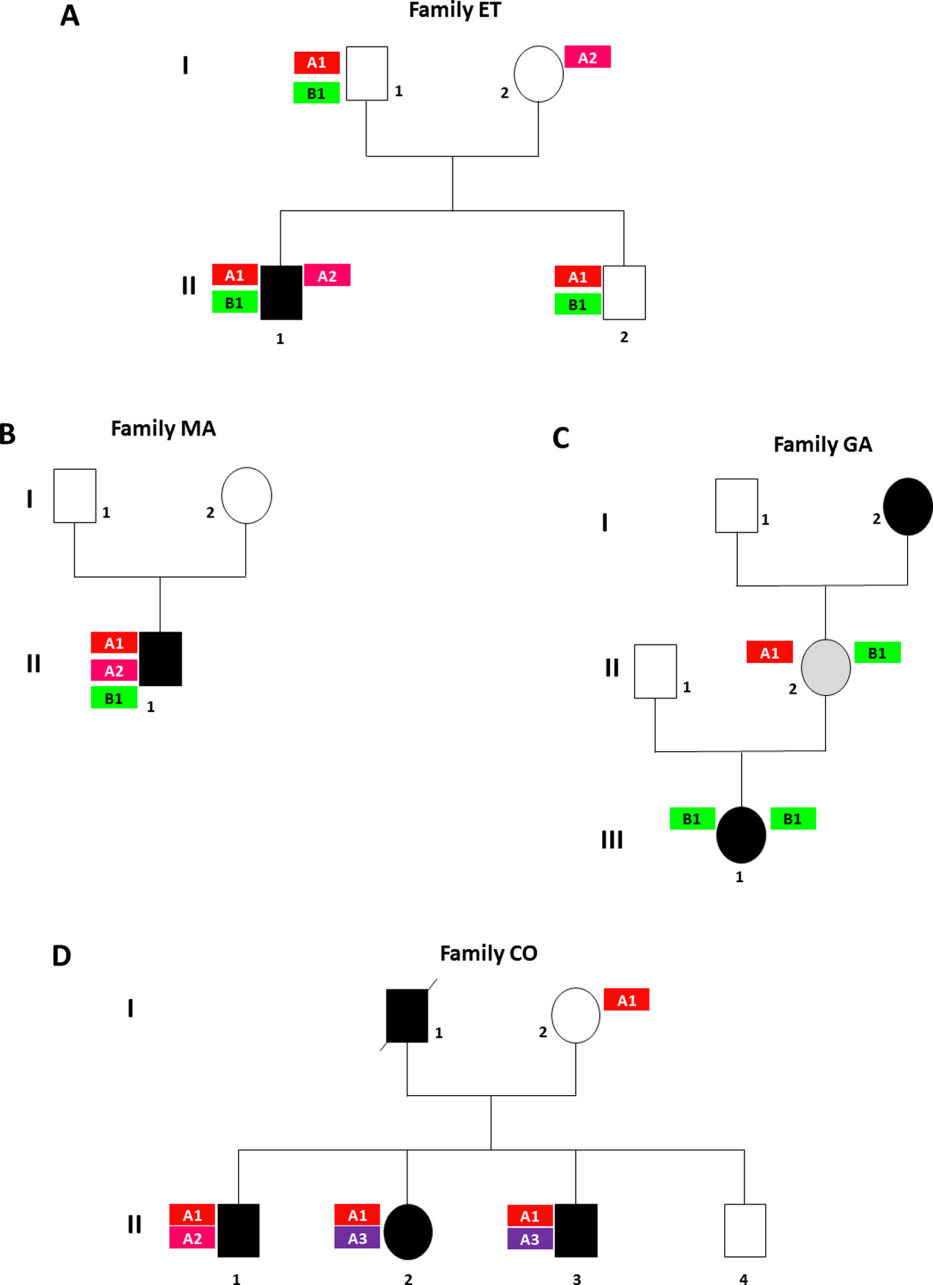
Genealogic trees of four families. (A) *Family ET*. A1 allele: c.163C>T – p.(Gln55*); A2 allele= c.(891+1_892‐1)_(1617+1_1618‐1)dup – p.(?); B1 allele= c.544G>A – p.(Ala182Thr). (B) *Family MA*. A1 allele: c.647C>T – p.(Thr216Met); A2 allele: c.(765+1_766‐1)_(1011+1_1012‐1)dup ‐ p.(Asp338Leufs*80); B1 allele: c.26G>A – p.(Arg9Gln). This missense variant is not predicted to be pathogenic (Table S3). (C) *Family GA*. A1 allele: c.566C>T – p.(Thr189Met); B1 allele: c.614dup – p.(Asn206Glufs*3). (D) *Family COL*. A1 allele: c.1500+1G>T – p.(?); A2 allele: *SLC3A1; NM_000341.3 :*c.(?_1)_*CAMKMT*; NM_024766.4 c.(311+1_?)del ‐ p.(?); A3 allele: c.1134C>A ‐ p.(Tyr378*). A denotes *SLC3A1* NM_000341; B denotes *SLC7A9* NM_014270.

Large‐scale rearrangement mapping allowed us to restore an accurate genotype in several families. Hence, QMPSF analyses were performed in patients in whom an AB genotype or a single variant has been identified after sequencing analyses. The identification of these rearrangements enabled us to discard an AB genotype being enough to explain full‐blown cystinuria. To illustrate these important findings, we detail the genotype and phenotype of three families (Fig. 3).

#### Family ET

The propositus P7 (Fig. 3A, II‐1) was diagnosed with cystinuria after an antenatal hyperechogenic colon had been identified through ultrasound during pregnancy. His cystinuria was elevated (471 μmol/mmol creatinine) but he never developed urinary stones up to the age of 5 years. The first mutated allele identified was [A1 = c.163C>T; p.(Gln55*)], but no other second *SLC3A1* variant was found. We then sequenced the *SLC7A9* gene and identified a heterozygote variant [B1 = c.544G>A; p.(Ala182Thr)] suggesting an AB genotype. Subsequently, QMPSF identified a second *SLC3A1* mutated allele (A2 = c.(891+1_892‐1)_(1617+1_1618‐1)dup) eventually proving genotype AAB. Interestingly, his brother (II‐2), 1 year older, has an A1B1 genotype and has a normal cystine excretion (2 μmol/mmol creatinine). Similarly, his father (I‐1) bearing the A1B1 genotype is asymptomatic but cystinuria data was unavailable. The mother, heterozygote for A2, was also asymptomatic.

#### Family MA

A 25‐year‐old man, P21 (Fig. 3B, II‐1) was diagnosed with cystinuria after he passed a urinary stone. He was born from nonconsanguineous parents who were both asymptomatic. His genotype was at first thought to be AO (A1 = c.647C>T; p.Thr216Met). We then sequenced *SLC7A9* and found one missense variant (B1 = c.26G>A; p.Arg9Gln) which was predicted to be nondeleterious (Table S3). Subsequently, QMPSF analysis revealed a second mutated allele in *SLC3A1* (A2 = c.(765+1_766‐1)_(1011+1_1012‐1)dup). Thus, his genotype was AA.

#### Family GA

The propositus P76 (Fig. 3C, III‐1) is an 11‐year‐old girl diagnosed with cystinuria after presenting with gross hematuria. Direct sequencing identified a homozygous BB genotype (B1 = c.614dup; pAsn206Glufs*3). Consanguinity was not known in this family. The father (II‐1) was asymptomatic. The mother (II‐2) did not suffer from urinary stones but her cystinuria was, however, in the high range (137 μmol/mmol creatinine). Besides the B1 allele, we identified a *SLC3A1* variant (A1 = c.566C>T; pThr189Met) in the mother, giving her an AB genotype.

Of note, the grandmother (I‐2) also suffered from typical cystine stone disease (diagnosed at the age of 16 years); unfortunately, her DNA was not available for analysis.

In addition, large‐scale rearrangement mapping enabled us to comprehensively interpret two different genotypes in affected siblings as described below.

#### Family CO

The propositus P70 (Fig. 3D, II‐1) was diagnosed with cystinuria after passing a renal stone at the age of 23 years. We identified a compound heterozygote *SLC3A1* variants A1 = c.1500+1G>T and A2 = *SLC3A1*: c.(?_1)_CAMKMT: c.(311+1_?)del. His sister (II‐2) also had typical cystine stone disease diagnosed at the age of 18 years (renal colic). Surprisingly, we did not identify the A2 allele but another A3 allele (c.1134C>A; pTyr378*). The brother (II‐3) was also affected and his genotype is A1/A3. Furthermore, the father (I‐1) had presented with cystine stones but was deceased and DNA not available for study. The mother (I‐2) was asymptomatic and her cystinuria was in the normal range (6 μmol/mmol). Her genotype was A1/O. Thus, the genotype of the deceased father was most certainly A2/A3.

### Clinical characterization

A cohort of 112 patients from 99 families was characterized and a questionnaire addressing, age at first symptoms, stone recurrence rates, metabolic evaluations, and follow‐up was completed. Sufficient data were provided for 109 patients belonging to 98 families (Table 4).

**Table 4:** Phenotype according to sex and genotype

		All (*n* = 109)	Male (*n* = 58)	Female (*n* = 51)	AA (*n* = 81)	BB (*n* = 24)
Age at first symptoms	Number	97	52	45	73	20
	Median	17 (0–57)	17 (0–57)	17 (2–40)	17 (0–57)	12 (1–40)^a^
Age at diagnosis	Number	98	51	48	76	19
	Median	18 (0–66)	21 (0–66)	18 (0–49)	21 (0–66)	14 (0–41)
Stone activity	Number	96	52	44	72	20
	Median	0.31 (0.00–2.67)	0.34 (0.00–2.67)	0.29 (0.00–2.09)	0.36 (0.00–2.67)	0.15 (0.00–1.25)
Follow‐up	Number	97	52	43	71	20
	Median	18 (1–59)	21 (1–59)	18 (1–52)	17 (1–52)	22 (1–59)
Cystinuria (μmol/mmol creat)	Number	89	48	41	65	21
	Median	249 (65–958)	218 (72–896)	290 (65–958)^b^	248 (65–958)	264 (72–597)
Age at last follow‐up	Median	36 (2–86)	37 (2–86)	35 (3–83)	36 (3–86)	38 (2–67)

a *P* = 0.04 (BB vs. AA).

b *P* = 0.004 (Females vs. Males).

The male/female ratio was 58 (53.2%)/51 (46.8%). Consanguinity was documented in 7/91 (7.7%) families. Eight out of 109 patients did not develop any symptoms due to their young age (the oldest being 8 years old) and/or to preventive therapy. Median age at first clinical event was 17 years (0 to 57) (mean 17 ± 2) and 44/101 patients (43.6%) had less than 15 years at first symptoms. For males, median age at first symptoms was 17 years (0 to 57) (mean age 15 ± 3), whereas it was 17 years (2 to 40) (mean age 17 ± 3) for females (*P* = 0.37). When comparing according to genotype, median age at first symptom was 17 years (0 to 57) (mean age 18 ± 2) for 74 patients A and 12 years (1 to 40) (mean age 13 ± 4) for 22 patients B (*P* = 0.04). Median age at diagnosis of cystinuria was 18 years (0 to 66) (mean age 20 ± 3). It was 21 years (0 to 66) (mean age 21 ± 4) for males and 18 years (0 to 49) (mean age 20 ± 3) for females.

Urinary cystine excretion at the time of diagnosis for 89 patients was, in median 249 μmol/mmol creatinine (65 to 958) (mean 285 ± 37). It was 218 μmol/mmol [72 to 896] (mean 245 ± 44) in males and 290 μmol/mmol [65 to 958] (mean 332 ± 59) in females (*P* = 0.004).

Patients with genotype A had median cystinuria excretion of 248 μmol/mmol [65 to 958] (mean 289 ± 46), whereas patients with genotype B had 264 μmol/mmol (72 to 597) (mean 278 ± 62) (*P* = 0.9).

Nephrolithiasis activity was assessed by the number of urological interventions (spontaneous stone emission rate was often lacking) related to the duration of the follow‐up, which was the period between the age at first symptoms and the age at the last follow‐up. Concerning surgical interventions, each patient had a median number of 5 (0 to 26) (mean 7 ± 1). During a median follow‐up of 18 years (1 to 59) (mean 22 ± 3), median urological intervention rate was 0.31/patient/year (0 to 2.67) (mean 0.46+/‐ 0.10). For males, it was 0.34/patient/year (0. to 2.67) (0.50 ± 0.14) and for females, 0.29/patient/year (0 to 2.09) (mean 0.41 ± 0.13) (*P* = 0.4). According to genotype, median intervention rates were 0.36/patient/year (0 to 2.67) (mean 0.50 ± 0.12) for patients A and 0.15/patient/year (0 to 1.25) (mean 0.36 ± 0.17) for patients B (*P* = 0.1).

Among patients above 15 years of age, 19 patients (21.8%) had chronic renal failure based on an eGFR <60 mL/mn (estimated by the MDRD). Among them, three patients had reached end stage renal failure at the age of 22, 24, and 35 years.

**Figure 4:**
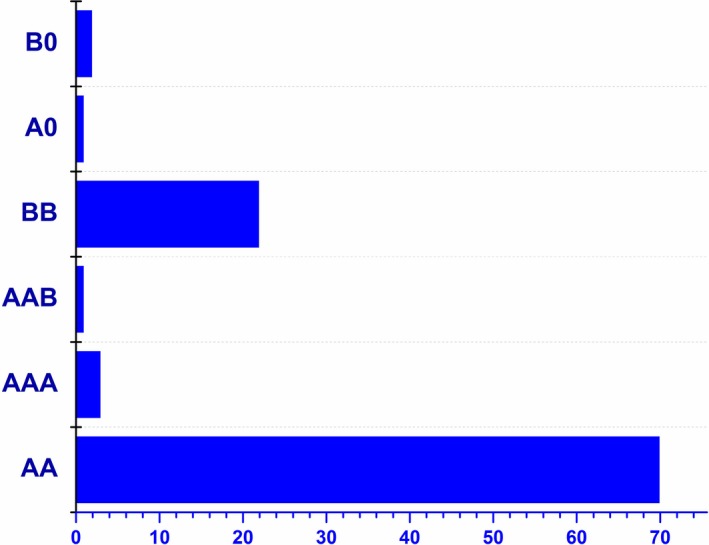
Genotype distribution of 99 patients. Seventy‐four patients with genotype A (70 AA, 3 AAA, 1 AAB), 22 with genotype B, 1 A0 and 2 B0 genotypes.

In summary, 88 pathogenic variants have been identified; 153 and 46 mutated alleles have been reported in *SLC3A1* and *SLC7A9* genes, respectively. Ninety‐six patients out of 99 were fully characterized at the molecular level, while in three subjects (P75, P98, P99) only one mutated allele was identified in *SLC7A9* or *SLC3A1* genes. It is worth noting that three mutated alleles have been characterized in four patients (three patients with genotype AAA, one patient with genotype AAB). Indeed, this study permits to classify the patients as follow: 74 patients having a genotype A (70 AA, 3 AAA, 1 AAB), 22 with genotype B, and 1 A0 and 2 B0 genotypes (Fig. 4). We did not identify AB genotype among the affected patients. Noticeably, two heterozygous variants in the *SLC3A1* and *SLC7A9* genes (c.566C>T – p.(Thr189Met) and c.614dup – p.(Asn206Glufs*3), respectively) were identified in a nonsymptomatic mother. Her daughter (P76) presented with cystinuria and a homozygous variant (c.614dup) was characterized (Table S3).

## Discussion

In this study, 112 cystinuric patients and 25 relatives from 99 families were screened for genomic variants of the *SLC3A1* and *SLC7A9* genes. We identified 88 pathogenic nucleotide changes of which 42 were novel. Recent studies reported an equal distribution of genotype A and B in European populations (Table S4). In our cohort, genotype A is more frequent than genotype B (*P* < 0.0001); indeed, 74 patients out of 99 harbor genotype A (70 AA, 3 AAA, 1 AAB), while 22 patients have a genotype B. Only three patients were not fully characterized (1A0 and 2 B0) which allows us to reach a detection rate of 97% in our cohort. Although disease variants are distributed uniformly among regions within *SLC3A1* and *SLC7A9* genes, some of them are more common in specific populations (Chillaron et al. 2010). We compared the frequency of common variants reported in a meta‐analysis (Chillaron et al. 2010) to that of our cohort (Table S5). No significant difference was observed for p.(Thr216Met), p.(Arg270*) variants in *SLC3A1* gene and p.(Gly105Arg), p.(Asn206Glufs*3), p.(Arg333Trp) variants in *SLC7A9* gene. The missense p.(Met467Thr) and the duplication c.(891+1_892‐1)_(1617+1_1618‐1)dup – p.(?) variants in *SLC3A1* gene are present with, respectively, a lower (*P* = 0.01) and a higher (*P* = 0.002) prevalence in the French cohort; the p.(Pro482Leu) variant in *SLC7A9* gene was not found in our cohort.

In an attempt to correlate genotypes and phenotypes, we compared the disease activity in patients with two missense variants (denoted as M), and patients with two severe variants (large deletion, duplication, small deletion/insertion, nonsense, splicing variants) denoted as S, in either *SLC3A1* or *SLC7A9*. Overall, 44 patients had M/M genotype and 27 patients had an S/S genotype. No significative differences were observed regarding age at first symptoms, age at diagnosis, stone activity, and urinary cystine excretion.

In this study, female patients have significantly higher urinary excretion of cystine compared to male patients, while no significant difference was observed in stone activity rate. Interestingly, Dello Strologo et al. reported that type B female patients had higher urinary excretion of cystine while male patients had on average a higher stone event rate than female patients (Dello Strologo et al. 2002).

Six cases with an antenatally diagnosed hyperechogenic colon were included in this study. No specific genotype was associated to this very early manifestation of cystinuria. Indeed, the genotype was AA in 4/6 (P2, P6, P20, P23), AAB in 1/6 (P7), and BB in 1/6 (P77). Only one study reported molecular characterization of a case presented with hyperechogenic colon; a homozygous variant in *SLC3A1* gene (*SLC3A1* NM_000341.3:c.833T>C – p.(Phe278Ser) was identified (Buxmann et al. 2014). The lack of genetic testing data impedes the correlation between the prenatal finding and the corresponding genotype. To the best of our knowledge, this is the first report of molecular characterization of a series of prenatal cystinuria.

Importantly, this study shows how the experimental assessment of the impact on splicing of *SLC3A1* and *SLC7A9* variants contributed to the full molecular characterization of cystinuria. Previously, in *SLC3A1* and *SLC7A9* genes, a very limited number of variants have been experimentally tested for their impact on splicing, using RT‐PCR analysis of patient RNA (Pras et al. 1998; Font‐Llitjos et al. 2005; Barbosa et al. 2011; Kummer et al. 2014). Here, in addition to four variants located in the most conserved positions of the splice sites (i.e., −2, −1/+1,+2), we identified using splicing minigene assays, six new splicing mutations. These variants have been classified into two groups based on their effects. The first group of splicing mutations alters directly the reference 5′ splice sites resulting into total or partial exon skipping. It includes two intronic variants and one synonymous variant located at the last base of the exon. The effects on splice sites of these three variants were correctly predicted by the in silico tool MaxEntSan. These data confirm the good performance of this type of bioinformatics prediction, as previously shown, in particular for large datasets of *BRCA1* and *BRCA2* variants (Houdayer et al. 2012).

More surprisingly, the second group of splicing mutations identified in this study induces exon skipping without alteration of the splice sites. It included two synonymous and one missense variants, located at distance from the splice sites. Their effects on splicing are mediated through the potential modification of exonic splicing regulatory elements (ESR). These 6–8 nucleotide sequences correspond to binding sites for splicing activator (exonic splicing enhancer ‐ ESE) or splicing repressor proteins (exonic splicing silencer ‐ ESS), respectively, which modulate the recruitment and activity of the splicing machinery. The three studied variants could either destroy ESE and/or create ESS. Interestingly, two of the splicing mutations, *SLC7A9* c.120G>A ‐ p.(=) and c.209C>T ‐ p.(Ala70Val), were identified in trans in a patient (P96) and both induce exon 3 skipping. Of note, the c.209C>T missense variant was supposed to induce a very mild decrease in L‐cystine transport activity (78% of wild‐type transport activity), as previously shown in functional protein assay (Font et al. 2001). But, as for the synonymous variant, we showed that this missense variant would be pathogenic through an impact on splicing regulation which would result onto a frameshift. To the best of our knowledge, in the literature, there is only one variant in *SLC7A9* gene reported to be responsible for a splicing defect through the alteration of ESR (Kummer et al. 2014). Interestingly, this variant, c.225C>T ‐ p.(=), is also located in *SLC7A9* exon 3, which suggest that the splicing regulation of this exon might be particularly sensitive to nucleotide changes. Based on this hypothesis, we performed bioinformatics predictions that could apprehend their effects on ESR. For this purpose, we used a recently developed method based on the reported quantitative evaluation of all RNA hexamers as potential ESR (Ke et al. 2011), which allows the calculation of total ESRseq score change (ΔtESRseq) for each variant, as previously described (Di Giacomo et al. 2013; Soukarieh et al. 2016). A good correlation has been previously described between the ΔtESRseq values and the experimental minigene data obtained for two large sets of variants located either in *BRCA2* exon 7 (Di Giacomo et al. 2013) or in *MLH1* exon 10 (Soukarieh et al. 2016). Interestingly, the three *SLC7A9* variants which experimentally induced exon 3 skipping are associated with negative ΔtESRseq values (Table 3), predictive of ESE loss and/or ESS gain. These data confirm the predictive power of this new *in silico* approach for the detection of ESR mutations. Of note, in the patient P97, two sequence variants were identified in the *SLC7A9* gene: one deleterious missense variant c.1166C>T p.(Thr389Met) and one synonymous nucleotide change, c.1032C>T p.(=), with unknown functional effect. Our *in silico* analysis revealed that this variant is associated with a negative ΔtESRseq value, predictive of a perturbation on splicing regulation. Indeed, the splicing minigene data confirmed that this variant induces the complete skipping of exon 10 which would result into a pathogenic frame shift. It would be of importance to confirm all these splicing defects from patient RNA but these samples were not available.

Altogether, these results suggest that the number of splicing mutations in *SLC3A1* and *SLC7A9* genes, especially those resulting into ESR alterations, might be currently underestimated. It has been estimated that approximately one third of all disease‐causing mutations alter splicing (Lim et al. 2011). Today, in diagnostic context, attention is given to intronic variants that could directly disturb reference splice sites. Moreover, special consideration should also be given to the potential splicing effects of exonic variants, whatever their predicted consequences at the protein level (missense, synonymous, or even nonsense). This study confirms that bioinformatics approaches dedicated to splice sites detection are useful for the identification of potential splicing mutations and, more importantly, it also extends the evaluation of a new *in silico* method based on ΔtESRseq for the detection of potential ESR mutations. This prediction method could represent a promising tool for selecting potential ESR‐alterating splicing mutations among a large number of variants before functional analysis. This strategy should improve the molecular diagnosis of cystinuria by uncovering the potential pathogenic nature of certain variants of unknown significance (VUS), through the demonstration of their impact on splicing.

Besides, large‐scale rearrangements play a significant role in the etiology of the disease; large‐scale deletions on chromosome 2p21 may include *SLC3A1*,* PERPL*, and *CAMKMT* genes. Moreover, duplications in *SLC3A1* gene and deletions in *SLC7A9* gene were characterized. Thus, QMPSF analyses allowed the detection of 37 mutated alleles (18% of mutant alleles).

The absence of AB genotype in recent studies and the identification of an AB genotype in nonsymptomatic relatives questioned the existence of the genotype AB (Chatzikyriakidou et al. 2008; Bisceglia et al. 2010). Indeed, no genotype AB has been identified in this study. Although two heterozygous variants in *SLC3A1* and *SLC7A9* genes, respectively, were characterized in two cystinuric patients [P7 and P21 (Table S3)], QMPSF analyses of *SLC3A1* gene allowed the identification of large duplications enabling to classify them as genotype A. In addition, genotype AB was identified in two nonsymptomatic relatives from two different families. These findings strongly support that digenic inheritance is unlikely. Besides, considering the high frequency of large‐scale deletion *SLC3A1* gene, we decided to perform QMPSF analysis in the 17 patients for whom a homozygous missense/nonsense variant was identified. Indeed, in three patients, P30, P33, and P39, QMPSF analysis allowed the identification of a deletion of the same region in which the missense/nonsense variant is located.

A part of negative genetic testing of cystinuria could be due to large genomic rearrangements that are missed by direct sequencing. Of note, two patients presented with Hypotonia‐Cystinuria Syndrome ‐ HCS (P31 and P72, Table S3) and suffered from neonatal hypotonia, growth retardation in addition to cystinuria. As in all HCS cases, our patients were homozygous for large‐scale 2p21 deletions. These variants involved both *SLC3A1* and the neighbored gene *PREPL* [*SLC3A1* NM_000341.3:c.(430+1_431‐1)_*PREPL* NM_006036.4:c.(219+1_?)del and *SLC3A1* NM_000341.3:c.(?_892‐1)_*PREPL*; NM_006036.4:c.(1896+1_?)del] in P31 and P72, respectively.

The breakpoints of these large‐scale variants may correspond to a high frequency of repetitive sequences within the *SLC3A1*,* PREPL*, and *CAMKMT* genes. Regions with high repeat sequence content are prone to nonallelic homologous recombination. RepeatMasker analysis showed that *SLC3A1*,* PREPL*, and *CAMKMT *genes contain a total of interspersed repeats in intronic regions corresponding, respectively, to 51.4%, 38%, and 46.5% of the gene sequence (Tarailo‐Graovac and Chen 2009). Indeed, Bisceglia et al. reported that the breakpoint of exons 2‐4 deletion involves Alu repeats at 227 bp upstream of exon 2 and 11680 bp downstream of exon 4 (c.431‐227_891+11680del17349) (Bisceglia et al. 2010). Besides, Schmidt et al. proposed that the duplication of exon 5 to exon 9 might be mediated by an unequal crossing‐over between intron 9 and intron 4 homologous regions (Schmidt et al. 2003).

In conclusion, this is the first study that investigated the molecular basis of cystinuria in French patients and the present report highlights that systematic screening for large‐scale rearrangements and specific investigations for the detection of splicing mutations are critical for the molecular characterization of cystinuria.

## Conflict of Interest

The authors have stated that they had no interests that might be perceived as posing a conflict or bias.

## Supporting information


**Table S1.** Nonpathogenic variants with an allele frequency >1%.

**Table S2.** In silico predictions for novel missense variants.

**Table S3.** Patient sequence variations.

**Table S4.** Genotype distribution in Europe.

**Table S5.** Comparison of recurrent mutation frequency.
