# Supplementary material for: Clinical and molecular characterization of cystinuria in a French cohort: relevance of assessing large‐scale rearrangements and splicing variants
Source: Mol Genet Genomic Med. 2017 May 16;5(4):373–89. doi: 10.1002/mgg3.294 (PMC5511796; doi:10.1002/mgg3.294)
Supplement: Supplementary file 1 — Table S1. Nonpathogenic variants with an allele frequency >1%. [file MGG3-5-373-s001.docx]

| **Supplemental Table 1: Non pathogenic variants with an allele frequency > 1%** | | | | |  | |  |
| --- | --- | --- | --- | --- | --- | --- | --- |
|  |  |  |  |  | |  |  |
| **Gene** | **Variant** | **dbSNP (rs)** | **1000 Genomes Frequency (%)** | **ExAC, Eur. (non-Finnish) Frequency (%)** | |  |  |
| ***SLC3A1*** | c.114A>C (p.=) | rs3738985 | 70.35 | 77,08 | |  |  |
|  | c.1136+3del (p.?) | rs61179824 | 11.18 | 10,28 | |  |  |
|  | c.1332+7C>T (p.?) | rs3738984 | 45.81 | 67,02 | |  |  |
|  | c.1854G>A (p.Met618Ile) | rs698761 | 46.09 | 68,11 | |  |  |
|  | c.*131T>C (p.?) | rs8886 | 42.45 | _ | |  |  |
|  |  |  |  |  | |  |  |
| ***SLC7A9*** | c.-323T>G (p.?) | rs2161518 | 26.34 | _ | |  |  |
|  | c.-172T>A (p.?) | rs2287876 | 46.03 | _ | |  |  |
|  | c.-39C>T (p.?) | rs11084677 | 16.95 | 6,54 | |  |  |
|  | c.235+22T>G (p.?) | rs12150904 | 28.69 | 34,35 | |  |  |
|  | c.236-112G>A (p.?) | rs12151145 | 7.21 | _ | |  |  |
|  | c.399C>T (p.=) | rs35170371 | 21.15 | 20,11 | |  |  |
|  | c.411T>C (p.=) | rs12150890 | 7.23 | 13,85 | |  |  |
|  | c.425T>C (p.Val142Ala) | rs12150889 | 30.61 | 34,57 | |  |  |
|  | c.478+10T>C (p.?) | rs6510300 | 8.09 | 14,38 | |  |  |
|  | c.507C>T (p.=) | rs11084673 | 28.00 | 34,18 | |  |  |
|  | c.604+10G>A (p.?) | rs11084672 | 7.23 | 13,89 | |  |  |
|  | c.604+24T>C (p.?) | rs11084671 | 28.02 | 34,17 | |  |  |
|  | c.667C>A (p.Leu223Met) | rs1007160 | 27.82 | 33,66 | |  |  |
|  | c.687C>T (p.=) | rs1007161 | 27.82 | 33,68 | |  |  |
|  | c.874-39A>G (p.?) | rs2287879 | 23.22 | 61,99 | |  |  |
|  | c.977+51A>G (p.?) | rs2287880 | 12.5 | 14,7 | |  |  |
|  | c.978-121C>T (p.?) | rs1559225 | 22.76 | _ | |  |  |
|  | c.1143C>T (p.=) | rs2287881 | 24.82 | 19,31 | |  |  |
|  | c.*79T>C (p. ?) | rs2287884 | 17.49 | _ | |  |  |
