# Supplementary material for: Clinical and molecular characterization of cystinuria in a French cohort: relevance of assessing large‐scale rearrangements and splicing variants
Source: Mol Genet Genomic Med. 2017 May 16;5(4):373–89. doi: 10.1002/mgg3.294 (PMC5511796; doi:10.1002/mgg3.294)
Supplement: Supplementary file 2 — Table S2. In silico predictions for novel missense variants. [file MGG3-5-373-s002.pdf]

Supplemental Table 2: In silico predictions for novel missense variants

Gene	Position	Nucleotide change	Predicted protein	Align GVGD	SIFT	MutationTaster	PolyPhen2
SLC3A1	Exon 1	c.257G>A	p.(Arg86Gln)	C0 (GV: 256.10 - GD: 0.00)	Tolerated (score: 0.19, median: 3.32)	disease causing (p-value: 1)	PROBABLY DAMAGING with a score of 1.000 (sensitivity: 0.00; specificity: 1.00)
	Exon 2	c.503C>T	p.(Ser168Leu)	C65 (GV: 0.00 - GD: 144.08)	Deleterious (score: 0, median: 3.01)	disease causing (p-value: 1)	PROBABLY DAMAGING with a score of 1.000 (sensitivity: 0.00; specificity: 1.00)
	Exon 2	c.595G>C	p.(Ala199Pro)	C0 (GV: 130.02 - GD: 1.62)	Tolerated (score: 0.23, median: 3.01)	disease causing (p-value: 0.988)	PROBABLY DAMAGING with a score of 0.992 (sensitivity: 0.70; specificity: 0.97)
	Exon 3	c.647C>G	p.(Thr216Arg)	C0 (GV: 223.27 - GD: 67.21)	Deleterious (score: 0.02, median: 3.01)	disease causing (p-value: 1)	PROBABLY DAMAGING with a score of 0.986 (sensitivity: 0.74; specificity: 0.96)
	Exon 3	c.763T>G	p.(Trp255Gly)	C0 (GV: 268.54 - GD: 58.26)	Deleterious (score: 0, median: 3.01)	disease causing (p-value: 1)	PROBABLY DAMAGING with a score of 0.993 (sensitivity: 0.70; specificity: 0.97)
	Exon 4	c.789T>G	p.(Ser263Arg)	C0 (GV: 215.83 - GD: 67.21)	Deleterious (score: 0.02, median: 3.01)	disease causing (p-value: 0.996)	PROBABLY DAMAGING with a score of 0.992 (sensitivity: 0.70; specificity: 0.97)
	Exon 4	c.851A>G	p.(Asp284Gly)	C0 (GV: 213.16 - GD: 64.73)	Deleterious (score: 0, median: 3.01)	disease causing (p-value: 1)	PROBABLY DAMAGING with a score of 0.993 (sensitivity: 0.70; specificity: 0.97)
	Exon 6	c.1051A>G	p.(Thr351Ala)	C0 (GV: 241.08 - GD: 0.00)	Tolerated (score: 0.07, median: 3.01)	disease causing (p-value: 1)	PROBABLY DAMAGING with a score of 1.000 (sensitivity: 0.00; specificity: 1.00)
	Exon 7	c.1144G>T	p.(Gly382Trp)	C0 (GV: 237.94 - GD: 66.04)	Deleterious (score: 0.04, median: 3.01)	disease causing (p-value: 1)	PROBABLY DAMAGING with a score of 1.000 (sensitivity: 0.00; specificity: 1.00)
	Exon 7	c.1318T>C	p.(Trp440Arg)	C0 (GV: 273.48 - GD: 30.75)	Deleterious (score: 0.02, median: 3.01)	disease causing (p-value: 1)	PROBABLY DAMAGING with a score of 0.995 (sensitivity: 0.68; specificity: 0.97)
	Exon 9	c.1518G>C	p.(Lys506Asn)	C0 (GV: 241.65 - GD: 4.86)	Deleterious (score: 0.02, median: 3.01)	disease causing (p-value: 0.999)	PROBABLY DAMAGING with a score of 0.992 (sensitivity: 0.70; specificity: 0.97)
	Exon 9	c.1527G>A	p.(Met509Ile)	C0 (GV: 245.76 - GD: 0.00)	Tolerated (score: 0.1, median: 3.01)	disease causing (p-value: 1)	PROBABLY DAMAGING with a score of 0.973 (sensitivity: 0.77; specificity: 0.96)
	Exon 9	c.1529A>C	p.(Gln510Pro)	C0 (GV: 226.93 - GD: 0.00)	Tolerated (score: 0.22, median: 3.01)	disease causing (p-value: 1)	POSSIBLY DAMAGING with a score of 0.927 (sensitivity: 0.81; specificity: 0.94)
SLC7A9	Exon 2	c.26G>A	p.(Arg9Gln)	C0 (GV: 244.67 - GD: 0.00)	Tolerated (score: 0.44, median: 3.32)	polymorphism (p-value: 0.575)	BENIGN with a score of 0.090 (sensitivity: 0.93; specificity: 0.85)
	Exon 4	c.380T>C	p.(Ile127Thr)	C65 (GV: 4.86 - GD: 89.28)	Deleterious (score: 0.01, median: 3.32)	disease causing (p-value: 1)	PROBABLY DAMAGING with a score of 0.992 (sensitivity: 0.70; specificity: 0.97)
	Exon 4	c.397T>C	p.(Ser133Pro)	C0 (GV: 103.39 - GD: 1.70)	Tolerated (score: 0.05, median: 3.32)	disease causing (p-value: 0.996)	BENIGN with a score of 0.408 (sensitivity: 0.89; specificity: 0.90)
	Exon 5	c.511C>G	p.(Arg171Gly)	C0 (GV: 98.89 - GD: 58.93)	Deleterious (score: 0.02, median: 3.32)	disease causing (p-value: 0.996)	POSSIBLY DAMAGING with a score of 0.687 (sensitivity: 0.86; specificity: 0.92)
