# Supplementary material for: Clinical and molecular characterization of cystinuria in a French cohort: relevance of assessing large‐scale rearrangements and splicing variants
Source: Mol Genet Genomic Med. 2017 May 16;5(4):373–89. doi: 10.1002/mgg3.294 (PMC5511796; doi:10.1002/mgg3.294)
Supplement: Supplementary file 3 — Table S3. Patient sequence variations. [file MGG3-5-373-s003.pdf]

Supplemental Table 3: Patient sequence variations.

Patient ID	Allele 1			Allele 2			Allele 3			Genotype
	Gene	Nucleotide change	Predicted Protein	Gene	Nucleotide change	Predicted Protein	Gene	Nucleotide change	Predicted Protein	
1	*SLC3A1*	c.647C>T	p.(Thr216Met)	*SLC3A1*	c.647C>T	p.(Thr216Met)				AA
2	*SLC3A1*	c.647C>T	p.(Thr216Met)	*SLC3A1*	c.647C>T	p.(Thr216Met)				AA
3	*SLC3A1*	c.1400T>C	p.(Met467Thr)	*SLC3A1*	c.789T>G	p.(Ser263Arg)				AA
4	*SLC3A1*	c.808C>T	p.(Arg270*)	*SLC3A1*	c.808C>T	p.(Arg270*)				AA
5	*SLC3A1*	c.647C>T	p.(Thr216Met)	*SLC3A1*	c.1400T>C	p.(Met467Thr)				AA
6	*SLC3A1*	c.1699_1700del	p.(Arg567Glyfs*9)	*SLC3A1*	c.2011C>T	p.(Arg671*)				AA
7	*SLC3A1*	c.163C>T	p.(Gln55*)	*SLC3A1*	c.(891+1_892-1)_(1617+1_1618-1)dup	p.(?)	*SLC7A9*	c.544G>A	p.(Ala182Thr)	AAB
8	*SLC3A1*	c.1159_1160delinsAA	p.(Ala387delinsLys)	*SLC3A1*	c.(430+1_431-1)_PREPL NM_006036.4:c.(219+1_?)del	p.(?)	*SLC3A1*	c.1607T>G	p.(Val536Gly)	AAA
9	*SLC3A1*	c.1051A>G	p.(Thr351Ala)	*SLC3A1*	c.1400T>C	p.(Met467Thr)				AA
10	*SLC3A1*	c.1318T>C	p.(Trp440Arg)	*SLC3A1*	c.1318T>C	p.(Trp440Arg)				AA
11	*SLC3A1*	c.371A>G	p.(Tyr124Cys)	*SLC3A1*	c.1640C>G	p.(Ser547Trp)				AA
12	*SLC3A1*	c.595G>C	p.(Ala199Pro)	*SLC3A1*	c.(?_1137-1)_PREPL NM_006036.4:c.(219+1_?)del	p.(?)				AA
13	*SLC3A1*	c.1364C>T	p.(Ser455Leu)	*SLC3A1*	c.(891+1_892-1)_(1617+1_1618-1)dup	p.(?)				AA
14	*SLC3A1*	c.594dup	p.(Ala199Serfs*4)	*SLC3A1*	c.808C>T	p.(Arg270*)				AA
15	*SLC3A1*	c.430+1G>T [1]	p.(?) [3]	*SLC3A1*	c.792G>A	p.(Trp264*)				AA
16	*SLC3A1*	c.647C>T	p.(Thr216Met)	*SLC3A1*	c.647C>T	p.(Thr216Met)				AA
17	*SLC3A1*	c.1144G>T	p.(Gly382Trp)	*SLC3A1*	c.(891+1_892-1)_(1617+1_1618-1)dup	p.(?)				AA
18	*SLC3A1*	c.1400T>C	p.(Met467Thr)	*SLC3A1*	c.1744_1745insC	p.(Tyr582Serfs*28)				AA
19	*SLC3A1*	c.1367G>A	P.(Arg467His)	*SLC3A1*	c.1367G>A	p.(Arg456His)				AA
20	*SLC3A1*	c.535G>T	p.(Asp179Tyr)	*SLC3A1*	c.1354C>T	p.(Arg452Trp)				AA
21	*SLC3A1*	c.647C>T	p.(Thr216Met)	*SLC3A1*	c.(765+1_766-1)_(1011+1_1012-1)dup	p.(?)				AA
22	*SLC3A1*	c.647C>T	p.(Thr216Met)	*SLC3A1*	c.647C>T	p.(Thr216Met)				AA
23	*SLC3A1*	c.1750del	p.(Arg584Glufs*14)	*SLC3A1*	c.(891+1_892-1)_(1617+1_1618-1)dup	p.(?)				AA
24	*SLC3A1*	c.566C>T	p.(Thr189Met)	*SLC3A1*	c.1400T>C	p.(Met467Thr)				AA
25	*SLC3A1*	c.566C>T	p.(Thr189Met)	*SLC3A1*	c.979_980del	p.(Asp327*)	*SLC3A1*	c.1699_1700del	p.(Arg567Glyfs*9)	AAA
26	*SLC3A1*	c.1400T>C	p.(Met467Thr)	*SLC3A1*	c.1684G>C	p.(Glu562Gln)	*SLC3A1*	c.1701G>T	p.(Arg567Ser)	AAA
27	*SLC3A1*	c.1400T>C	p.(Asp284Gly)	*SLC3A1*	c.1400T>C	p.(Met467Thr)				AA
28	*SLC3A1*	c.851A>G	p.(Asp284Gly)	*SLC3A1*	c.1617+5G>A [2]	p.(?) [3]				AA
29	*SLC3A1*	c.808C>T	p.(Arg270*)	*SLC3A1*	c.808C>T	p.(Arg270*)				AA
30	*SLC3A1*	c.1134C>A	p.(Tyr378*)	*SLC3A1*	c.(430+1_431-1)_PREPL NM_006036.4:c.(219+1_?)del	p.(?)				AA
31	*SLC3A1*	c.(430+1_431-1)_PREPL NM_006036.4:c.(219+1_?)del	p.(?)	*SLC3A1*	c.(430+1_431-1)_PREPL NM_006036.4:c.(219+1_?)del	p.(?)				AA
32	*SLC3A1*	c.1459G>T	p.(Gly487*)	*SLC3A1*	c.(891+1_892-1)_(1617+1_1618-1)dup	p.(?)				AA
33	*SLC3A1*	c.808C>T	p.(Arg270*)	*SLC3A1*	c.(610+1_611-1)_(1616+1_1617-1)del	p.(Leu205Profs*22)				AA
34	*SLC3A1*	c.1529A>C	p.(Gln510Pro)	*SLC3A1*	c.(891+1_892-1)_(1617+1_1618-1)dup	p.(?)				AA
35	*SLC3A1*	c.1354C>T	p.(Arg452Trp)	*SLC3A1*	c.1400T>C	p.(Met467Thr)				AA
36	*SLC3A1*	c.1699_1700del	p.(Arg567Glyfs*9)	*SLC3A1*	c.(765+1_766-1)_(1616+1_1617-1)del	p.(Leu256_Pro337del)				AA
37	*SLC3A1*	c.503C>T	p.(Ser168Leu)	*SLC3A1*	c.1400T>A	p.(Met467Lys)				AA
38	*SLC3A1*	c.1094G>T	p.(Arg365Leu)	*SLC3A1*	c.1400T>C	p.(Met467Thr)				AA
39	*SLC3A1*	c.1400T>C	p.(Met467Thr)	*SLC3A1*	c.(1332+1_1333-1)_(1500+1_1501-1)del	p.(Ile445_Ile500del)				AA
40	*SLC3A1*	c.535G>T	p.(Asp179Tyr)	*SLC3A1*	c.1400T>C	p.(Met467Thr)				AA
41	*SLC3A1*	c.1400T>C	p.(Met467Thr)	*SLC3A1*	c.(891+1_892-1)_(1617+1_1618-1)dup	p.(?)				AA
42	*SLC3A1*	c.1400T>C	p.(Met467Thr)	*SLC3A1*	c.(891+1_892-1)_(1617+1_1618-1)dup	p.(?)				AA
43	*SLC3A1*	c.763T>G	p.(Trp255Gly)	*SLC3A1*	c.1400T>C	p.(Met467Thr)				AA
44	*SLC3A1*	c.647C>T	p.(Thr216Met)	*SLC3A1*	c.1400T>C	p.(Met467Thr)				AA
45	*SLC3A1*	c.1607T>G	p.(Val536Gly)	*SLC3A1*	c.1159_1160delinsAA	p.(Ala387delinsLys)				AA
46	*SLC3A1*	c.1500+1G>T [1]	p.(?) [3]	*SLC3A1*	c.1545_1563dup	p.(Ala522Lysfs*6)				AA
47	*SLC3A1*	c.1011G>A [2]	p.(Pro337=) [3]	*SLC3A1*	c.1400T>C	p.(Met467Thr)				AA
48	*SLC3A1*	c.1400T>C	p.(Met467Thr)	*SLC3A1*	c.(891+1_892-1)_(1617+1_1618-1)dup	p.(?)				AA
49	*SLC3A1*	c.(430+1_431-1)_(891+1_892-1)del	p.(Ile145Asnfs*11)	*SLC3A1*	c.(?_1618-1)_PREPL NM_006036.4:c.(219+1_?)del	p.(?)				AA
50	*SLC3A1*	c.1400T>A	p.(Met467Lys)	*SLC3A1*	c.(891+1_892-1)_(1617+1_1618-1)dup	p.(?)				AA
51	*SLC3A1*	c.1011G>A [2]	p.(Pro337=) [3]	*SLC3A1*	c.1190A>G	p.(Tyr397Cys)				AA
52	*SLC3A1*	c.1400T>C	p.(Met467Thr)	*SLC3A1*	c.(891+1_892-1)_(1617+1_1618-1)dup	p.(?)				AA
53	*SLC3A1*	c.647C>G	p.(Thr216Arg)	*SLC3A1*	c.1500+1G>T [1]	p.(?) [3]				AA
54	*SLC3A1*	c.763T>G	p.(Trp255Gly)	*SLC3A1*	c.1400T>C	p.(Met467Thr)				AA
55	*SLC3A1*	c.257G>A	p.(Arg86Gln)	*SLC3A1*	c.(891+1_892-1)_(1617+1_1618-1)dup	p.(?)				AA
56	*SLC3A1*	c.464T>G	p.(Leu155*)	*SLC3A1*	c.647C>T	p.(Thr216Met)				AA
57	*SLC3A1*	c.1518G>C	p.(Lys506Asn)	*SLC3A1*	c.1518G>C	p.(Lys506Asn)				AA
58	*SLC3A1*	c.1190A>G	p.(Tyr397Cys)	*SLC3A1*	c.1190A>G	p.(Tyr397Cys)				AA
59	*SLC3A1*	c.(891+1_-892-1)_(1617+1_1618-1)dup	p.(?)	*SLC3A1*	c.(891+1_892-1)_(1617+1_1618-1)dup	p.(?)				AA
60	*SLC3A1*	c.1352C>G	p.(Ser451*)	*SLC3A1*	c.1796T>C	p.(Phe599Ser)				AA
61	*SLC3A1*	c.1400T>C	p.(Met467Thr)	*SLC3A1*	c.(891+1_892-1)_(1617+1_1618-1)dup	p.(?)				AA
62	*SLC3A1*	c.647C>T	p.(Thr216Met)	*SLC3A1*	c.647C>T	p.(Thr216Met)				AA
63	*SLC3A1*	c.792G>A	p.(Trp264*)	*SLC3A1*	c.1400T>C	p.(Met467Thr)				AA
64	*SLC3A1*	c.1400T>C	p.(Met467Thr)	*SLC3A1*	c.1400T>C	p.(Met467Thr)				AA
65	*SLC3A1*	c.1400T>C	p.(Met467Thr)	*SLC3A1*	c.(891+1_892-1)_(1617+1_1618-1)dup	p.(?)				AA
66	*SLC3A1*	c.647C>T	p.(Thr216Met)	*SLC3A1*	c.647C>T	p.(Thr216Met)				AA
67	*SLC3A1*	c.1364C>T	p.(Ser455Leu)	*SLC3A1*	c.1527G>A	p.(Met509Ile)				AA
68	*SLC3A1*	c.1354C>T	p.(Arg452Trp)	*SLC3A1*	c.1400T>C	p.(Met467Thr)				AA
69	*SLC3A1*	c.1354C>T	p.(Arg452Trp)	*SLC3A1*	c.(891+1_892-1)_(1617+1_1618-1)dup	p.(?)				AA
70	*SLC3A1*	c.1500+1G>T [1]	p.(?) [3]	*SLC3A1*	c.(?_1)_CAMKMT; NM_024766.4 c.(311+1_?)del	p.(?)				AA
71	*SLC3A1*	c.808C>T	p.(Arg270*)	*SLC3A1*	c.1011G>A [2]	p.(Pro337=) [3]				AA
72	*SLC3A1*	c.(?_892-1)_PREPL; NM_006036.4:c.(1896+1_?)del	p.(?)	*SLC3A1*	c.(?_892-1)_PREPL; NM_006036.4:c.(1896+1_?)del	p.(?)				AA
73	*SLC3A1*	c.1754_1755del	p.(Glu585Alafs*24)	*SLC3A1*	c.(?_1618-1)_CAMKMT NM_024766.4:c.(376+1_?)del	p.(?)				AA
74	*SLC3A1*	c.1400T>C	p.(Met467Thr)	*SLC3A1*	c.(430+1_431-1)_(765+1_766-1)del	p.(Gly144Valfs*12				AA
75	*SLC7A9*	c.397T>C (VUS)	p.(Ser133Pro)	*SLC7A9*	c.992C>T	p.(Ala331Val)				B?
76	*SLC7A9*	c.614dup	pAsn206Glufs*3)	*SLC7A9*	c.614dup	p.(Asn206Glufs*3)				BB
77	*SLC7A9*	c.313G>A	p.(Gly105Arg)	*SLC7A9*	c.614dup	p.(Asn206Glufs*3)				BB
78	*SLC7A9*	c.562G>A	p.(Val188Met)	*SLC7A9*	c.(1224+1_1225-1)_(1399+1_1400-1)del	p.(Val409Serfs*3				BB
79	*SLC7A9*	c.313G>A	p.(Gly105Arg)	*SLC7A9*	c.313G>A	p.(Gly105Arg)				BB
80	*SLC7A9*	c.131T>C	p.(Ile44Thr)	*SLC7A9*	c.380T>C	p.(Ile127Thr)				BB
81	*SLC7A9*	c.313G>A	p.(Gly105Arg)	*SLC7A9*	c.313G>A	p.(Gly105Arg)				BB
82	*SLC7A9*	c.1393dup	p.(Ile465Asnfs*23)	*SLC7A9*	c.1399+3_1399+6del	p.(?)				BB
83	*SLC7A9*	c.313G>A	p.(Gly105Arg)	*SLC7A9*	c.414_415del	p.(Pro139Leufs*69)				BB
84	*SLC7A9*	c.313G>A	p.(Gly105Arg)	*SLC7A9*	c.508G>A	p.(Val170Met)				BB
85	*SLC7A9*	c.517G>C	p.(Gly173Arg)	*SLC7A9*	c.614dup	p.(Asn206Glufs*3)				BB
86	*SLC7A9*	c.511C>G	p.(Arg171Gly)	*SLC7A9*	c.1074+2T>C [1]	p.(?) [3]				BB
87	*SLC7A9*	c.368C>T	p.(Thr123Met)	*SLC7A9*	c.544G>A	p.(Ala182Thr)				BB
88	*SLC7A9*	c.313G>A	p.(Gly105Arg)	*SLC7A9*	c.614dup	p.(Asn206Glufs*3)				BB
89	*SLC7A9*	c.313G>A	p.(Gly105Arg)	*SLC7A9*	c.604+2T>C [1]	p.(?) [3]				BB
90	*SLC7A9*	c.285del	p.(Glu96Serfs*5)	*SLC7A9*	c.544G>A	p.(Ala182Thr)				BB
91	*SLC7A9*	c.313G>A	p.(Gly105Arg)	*SLC7A9*	c.313G>A	p.(Gly105Arg)				BB
92	*SLC7A9*	c.(977+1_978-1)_(1074+1_1075-1)del	p.(Leu327Valfs*3)	*SLC7A9*	c.(1224+1_1225-1)_(1399+1_1400-1)del	p.(Val409Serfs*3				BB
93	*SLC7A9*	c.91delinsTGTGAT	p.(Gly31Cysfs*61)	*SLC7A9*	c.313G>A	p.(Gly105Arg)				BB
94	*SLC7A9*	c.997C>T	p.(Arg333Trp)	*SLC7A9*	c.997C>T	p.(Arg333Trp)				BB
95	*SLC7A9*	c.313G>A	p.(Gly105Arg)	*SLC7A9*	c.992C>T	p.(Ala331Val)				BB
96	*SLC7A9*	c.120G>A [2]	p.(Val40=) [3]	*SLC7A9*	c.209C>T [2]	p.(Ala70Val) [3]				BB
97	*SLC7A9*	c.1032C>T [2]	p.(Ile344=) [3]	*SLC7A9*	c.1166C>T	p.(Thr389Met)				BB
98	*SLC7A9*	c.313G>A	p.(Gly105Arg)							B0
99	*SLC3A1*	c.1693_1695del	p.(Leu565del)							A0

[1]Variants located int the canonical intronic splice site sequences; [2]Variants with an impact on splicing in minigene assay (this study); [3]Predicted consequences at the protein level without taking into account the impact of the variant on splicing. VUS: Variant of Unknown Significance.
