# Supplementary material for: Clinical and molecular characterization of cystinuria in a French cohort: relevance of assessing large‐scale rearrangements and splicing variants
Source: Mol Genet Genomic Med. 2017 May 16;5(4):373–89. doi: 10.1002/mgg3.294 (PMC5511796; doi:10.1002/mgg3.294)
Supplement: Supplementary file 4 — Table S4. Genotype distribution in Europe. [file MGG3-5-373-s004.doc]

| **Year**  **(Reference)** | **Country** | **n** | **AA(%)** | **BB(%)** | **A0(%)** | **B0(%)** | **00(%)** | **% diagnostic** |
| --- | --- | --- | --- | --- | --- | --- | --- | --- |
| Bisceglia, et al., 2010 | Italy | 172 | 43,2 | 44,4 | 0,6 | 7,4 | 4,3 | 87.6 |
| Chatzikyriakidou, et al., 2008 | Greece | 52 | 53,8 | 34,6 | 0,0 | 1,9 | 9,6 | 88.4 |
| Wong, et al., 2015 | United Kingdom | 76 | 37 | 29 | 7 | 22 | 5 | 66 |
| This Study | France | 99 | 74 | 22 | 1 | 2 | 0 | 96.9 |

**Supplemental Table 4**. **Genotype distribution in Europe.**
