# Supplementary material for: Clinical and molecular characterization of cystinuria in a French cohort: relevance of assessing large‐scale rearrangements and splicing variants
Source: Mol Genet Genomic Med. 2017 May 16;5(4):373–89. doi: 10.1002/mgg3.294 (PMC5511796; doi:10.1002/mgg3.294)
Supplement: Supplementary file 5 — Table S5. Comparison of recurrent mutation frequency. [file MGG3-5-373-s005.doc]

**Supplemental Table 5: Comparison of recurrent mutation frequency**

| ***SLC3A1* Variants** | | **Allele frequency (mutated allele number)** | | ***p*** |
| --- | --- | --- | --- | --- |
|  |  | **Meta-analysis^$^**  (579 mutated alleles) | **This study**  (153 mutated alleles) |  |
| c.1400T>C | p.(Met467Thr) | 27.3 % (158) | 17.6 % (27) | **0 .01*** |
| c.647C>T | p.(Thr216Met) | 12.3 % (70) | 10.5 % (16) | 0.54 |
| c.892-?_1617+?dup | p.(Glu298_Asp539dup) | 4.7 % (27) | 11.8 % (18) | **0.02*** |
| c.808C>T) | p.(Arg270*) | 4.1 % (24) | 4.6 % (7) | 0.78 |
| ***SLC7A9* Variants** | | **Meta-analysis**  (436 mutated alleles) | **This study**  (48 mutated alleles) | ***p*** |
| c.313G>A | p.(Gly105Arg) | 21.1 % (92) | 29.1 % (14) | 0.16 |
| c.1445C>T | p.(Pro482Leu) | 13.1 % (57) | 0.0 % (0) | **0.008*** |
| c.614dup | p.(Asn206Glufs*3) | 6.7 % (29) | 10.9 % (5) | 0.32 |
| c.997C>T | p.(Arg333Trp) | 6.4 % (28) | 4.2 % (2) | 0.55 |

**p*<0.05

**^$^** Meta-analysis Chillaron, et al., 2010
